# Is It Safe Enough? An IPA Study of How Couple Therapists Make Sense of Their Decision to Either Stop or Continue with Couple Therapy When Violence Becomes the Issue

**DOI:** 10.3390/bs14010037

**Published:** 2024-01-05

**Authors:** Jan Frode Snellingen, Pål Erik Carlin, Arlene Vetere

**Affiliations:** 1 Centre for Diaconia and Professional Practice, VID Specialized University, P.O. Box 184 Vinderen, 0319 Oslo, Norway; pal.erik.carlin@bufetat.no; 2 Faculty of Social Studies, VID Specialized University, P.O. Box 184 Vinderen, 0319 Oslo, Norway; drarlenevetere@hotmail.com

**Keywords:** partner violence, couple violence, domestic violence, couple therapy, conjoint sessions, clinical sense making, qualitative method, IPA, therapist experience

## Abstract

Background: Couple therapists will encounter couple violence in their practice at some point. In this context, one of the main questions they must address is whether to continue with conjoint sessions. This study explores how couple therapists make sense of their decision whether or not to continue with conjoint sessions when violence has become an issue. Methods: This qualitative study used four semi-structured focus groups and Interpretative Phenomenological Analysis (IPA) to analyse the data from the twelve experienced couple therapist participants. Results: Our IPA analysis led to three main group experiential themes across the focus groups: (1) Is it safe enough? (2) Do we have a joint and regenerative project? (3) Three key sources for sense making. Conclusion: Partner violence challenges the realm of couple therapy. This article explored how the couple therapists orient themselves and grapple with decision making when violence becomes an issue. The article offers unique insights regarding what the therapists orient themselves towards and how they try to form an impression of whether to continue conjoint sessions. We outline immediate clinical implications and propose measures for building individual and organisational capacity regarding “clinical sense making”. Suggestions for further research are also addressed.

## 1. Introduction

*“At times, I get the same sensation as if I’m sitting in a packed conference hall with 5000 people. It’s that moment when you raise your hand to speak and that unmistakable feeling, that’s what I sense sometimes. It’s a profound discomfort. You feel it in your stomach, almost like a sensation of weightlessness, as just before you’re taking a leap. Because that’s what it feels like sometimes in our line of work. You’re taking a leap, making a choice. Because that’s the way it is. Sometimes you have to make a choice that you’re acutely aware that this may have consequences. That’s just the nature of our job”.*

(Stig, a participant therapist from focus group 4)

The quotation above illustrates why we wanted to do this research, exploring how couple therapists make sense of their decision as to whether or not they continue with the therapy when violent behaviour has become the issue. Couple and family therapists will encounter aggression and family violence in their practice at some point [1]. In this context, violence can be part of the referral or can become one of the main concerns at every stage of collaboration with the couple.

Prevalence surveys in Norway and internationally show that 14–30% of respondents in community samples state that they have been exposed to partner violence at some point in their life cycle [2,3,4,5]. In clinical populations, the prevalence of partner violence is even higher. For couples seeking couple and family therapy in generalist settings, studies show that as much as 70% of couples have experienced physical aggression and relational violence [6,7]. There have been a few research studies on different aspects of family violence in the Norwegian Family Counselling Services (NFCS) showing variation in the prevalence of family violence between 0.3 and 34% at various family counselling offices [8,9,10].

In parallel with the high prevalence of violent behaviour in couples seeking therapy, it has been controversial and repeatedly debated as to whether couple therapy is safe, appropriate, or even an ethical form of treatment for partner violence [11,12]. However, studies show that couple therapy can be appropriate and as effective as other interventions when both the violence and the couples are carefully screened and appropriately assessed for conjoint therapy [1,11,13,14]. Couple therapy can, through these measures, sometimes be considered safe enough and help to reduce violence and increase relationship satisfaction because some violent behaviours can be understood as the result of escalated interactional patterns born out of frustration, anxiety, and unmet attachment needs [15,16,17,18,19].

There is a growing recognition that violence in intimate relationships is not a uniform phenomenon but is better conceptualised along a multifactored continuum differentiated by the type of violence, degree of frequency, aspects of power and coercive control, degree of severity, the experience of those involved, gender and consequences [20,21,22,23,24]. Johnson’s conceptual framework [20,25,26,27] is frequently referred to for classifying types of couples’ violence. It distinguishes between two primary contexts: coercive controlling violence and situational couple violence. Coercive controlling violence typically unfolds within a framework of coercive control, where one partner repeatedly uses multiple coercive measures to control and dominate the other partner. In contrast, situational couple violence can be understood as the result of escalated conflicts where one or both partners contribute to the destructive interactional pattern. There have also been several efforts in the research and practice field to differentiate both the conceptualisation of and response to family violence [28,29,30]. These efforts emphasise the importance of assessment because the motivation, characteristics, and effects of couple violence vary widely across multiple spectrums of individual and interactional features. The therapist must gather information and consider these variations in their ongoing practice, response choice, and possible treatment.

In addition, addressing couples in therapy introduces distinctive ethical challenges not typically faced by individual therapists [31]. When violence becomes a concern, clinicians providing couple therapy are likely to face and grapple with heightened ethical and legal dilemmas throughout the treatment process, aiming to safeguard the well-being of each partner [32,33]. When ethical aspects are taken into account along with a thorough assessment, it can enhance treatment effectiveness and promote the right effort at the right time regarding partner violence.

Guidelines for clinical-based criteria have been developed for assessment of the possibility of whether couple therapy is an appropriate treatment modality [12,14,17,34]. These guidelines mostly emphasise structures, sequences and lists of criteria that outline the material content but pay less attention to the relational processes the therapists themselves navigate to that end. Emphasising the process of assessing for the appropriate treatment modality can be referred to, more generally, as a part of a formulation in psychotherapy. For example, Vetere [35] (p. 388) describes formulation as:

*“a crucial practice in psychotherapy, whereby explanations for experience are brought together with an evidence base for practice within an ethical framework of conduct. This guides and directs action for all participants within an iterative process of feedback and action. Formulation is emancipatory in intent, and provides accountability for practice.”*

A formulation will draw on theory and research but also on intuitive and tacit knowledge to provide a framework for describing a client’s problems, needs and resources. It can be a starting point for action and further investigation, such as progressive hypothesising in a collaborative process with the couple or the family [36]. So, this formulation process will transcend merely evaluating the presence or absence of violence. Therefore, when partner violence becomes an issue, couple and family therapists must have sufficient knowledge, skills, and resources to assess and make sense of the situation to choose an adequate response to the couple violence in question [37]. This process of evaluating and making sense of the situation is not confined to the initial phases after the violence has become a primary concern; it should be recognised as an ongoing and integral function that extends across all stages of the therapist’s engagement with the couple [17,18,34,38].

One of the main questions the therapist must address and make sense of when violence becomes an issue in couple therapy is whether or not to continue with conjoint sessions. This decision hinges on ensuring the safety and well-being of all parties involved while also striving to enable beneficial therapeutic outcomes. Currently, there is little research on how couple therapists go about their ‘natural’ clinical sense making when faced with the emergence of couple violence as the primary concern, particularly when they try to make sense of the situation and determine whether it is appropriate to proceed with conjoint sessions. So, a deeper understanding and knowledge of what plays a part in such decisions can aid couple and family therapists in enhancing safe practice and service provision and promoting the right effort at the right time in matters of couple violence. Thus, the questions addressed in this research include the following: What do the therapists base their decisions on? What influences their formulation and practice? How do they orient themselves, and what considerations are taken into account when deciding whether conjoint sessions are safe and relevant to continue with? The main research question was: How are couple therapists making sense of their decision whether or not to continue with conjoint sessions when violence has become an issue?

## 2. Methods

### 2.1. The Research Context

The research was conducted within the Norwegian Family Counselling Services (NFCS). The NFCS is a state-funded, non-referral and free service available to couples, non-cohabitating parents, and families struggling with family or relationship issues. The services are offered at 42 family counselling offices located throughout Norway, employing about 500 therapists (social workers, psychologists, and other couple and family therapy specialists). The primary mandate of the services is to strengthen the relational conditions of children’s development by supporting couple, family, and parental functioning. In 2022, the services worked with approximately 55,000 cases, including both therapy and mediation [39]. As a result, the NFCS is one of the services that has been given a more prominent role in addressing and preventing family violence, as families seeking couple and family therapy constitutes a population at risk for relational aggression and violence [40,41,42,43,44,45]. However, the NFCS is a generalist couple and family therapy service, with practitioners defining themselves as systemically and relationally oriented, and is not a specialised couple and family violence service. The therapists at the NFCS primarily utilise conjoint sessions when working with couples and families, although individual sessions are not uncommon. In cases where violence becomes an issue, there are few standardised procedures; however, individual sessions will often be incorporated strategically to address specific issues or as a part of the assessment and to ensure the well-being of each participant. The study was conducted amidst and following the COVID-19 pandemic, and it captures therapists’ experiences against the backdrop of the global crisis.

### 2.2. Design and Method

Qualitative research has much to offer psychotherapy researchers by generating new understanding of the complexity and experience of different participants in therapy [46]. Interpretative Phenomenological Analysis (IPA) was chosen as an inspiration for this study’s analytic method for the focus group data reported here. IPA’s analytical focus can be suitable for exploring how a phenomenon is experienced and understood by a specific group in a particular context [47,48]. We use the word “inspiration” in the sentence above because IPA offers flexible guidelines and should not be treated as a recipe but put to use flexibly and creatively according to the research project [48]. In a narrative review, Anderson, Slark and Gott [49] (p. 98) conclude that “IPA is a methodology ideally suited to exploration of decision making in complex real-world clinical contexts. A small corpus of IPA studies has produced valuable insights into a range of clinical decision making contexts”.

### 2.3. Data Collection

This study utilised four focus groups (FGs) to collect data. The application of IPA to focus group data is not simplistic, as focus groups “may be less obviously suitable for IPA researchers, but they have certainly been employed” [48] (p. 125). Several authors argue that the focus group interview makes it challenging to understand an individual’s subjective experience [50,51,52] and that focus groups enhance a collective group voice at the expense of the individual [53]. At the same time, the focus group setting also has some advantages. Focus groups can be helpful because they allow multiple voices to be heard, drawing on a larger sample but in a reduced number of settings for data collection [54]. In addition, the focus groups can inspire, scaffold, and help the participants to go deeper and illuminate both convergence and divergence in experiences, making even thicker descriptions of the phenomenon in question. Both first and second authors conducted all four focus groups together.

### 2.4. Participants and Recruitment

The recruitment was conducted through purposive sampling because the participants could illuminate and provide insight into the specific phenomenon of interest in a relevant context [55]. We chose a focus group format with a maximum of four participants. This was to achieve optimal depth and possible ideographic commitment in the interviews [48]. In total, 12 therapists from four different offices within the NFCS were recruited for the study. We also used existing collegial groups because that can help to provide the safety needed for in-depth exploring and reflecting in a shared discussion of sensitive experiences [48]. All therapists have education in couple and family therapy and at least a basic training working with couples where violence is an issue. They are also all involved in their respective family counselling offices’ “violence team”. This means the therapists use most of their role for “ordinary” couple and family work but use some of their position to strengthen the work in the office and work clinically in cases where violence becomes an issue. Otherwise, there is diversity regarding gender, age and duration of employment in the NFCS (see Table 1).

### 2.5. Analysis of the Focus Group Data

The essence of an Interpretative Phenomenological study lies in its analytical focus. The researcher’s commitment is to move from the descriptive to a more interpretative stance regarding the participants’ experience and meaning making of a phenomenon of interest in a specific context [48]. However, the existing literature on analyses in IPA has not prescribed a single “method” for working with the data. Nevertheless, some general processes and strategies within IPA are suggested and could be applied flexibly to optimise the given analytic task and study [48,55,56]. Several articles describe and demonstrate a step-by-step guide on how IPA can be adapted for use with focus groups [53,54,57]. Elements of these efforts were drawn upon when constructing our way forward in analysing the material with the following strategies/steps:

Step 1: Identifying the researcher’s orientation and potential bias

Before the initial focus group, we, the first and second authors engaged in a self-reflexive interview to illuminate our presumptions, biases, and potential influences on the research and analytical process [58]. Following each focus group, we conducted mutual interviews, recording the following. (1) What surprised us? (2) What were we drawn towards? (3) Was there anything we would like to do more or less of if we had the opportunity to do it again? Additionally, a reflective research diary was maintained throughout the study, capturing notes on challenges, dilemmas, decisions, insights, and positive experiences. These reflections were included in and influenced the next steps and iterative cycles of the further research process.

Step 2: Immersion in the data and exploratory noting

We started with the first focus group. The transcript was read through in its entirety without taking notes. Then, we re-read the interview while listening to the audiotape. Throughout re-reading and listening, exploratory notes were taken: spontaneous thoughts, ideas, feelings, etc. These notes made it somewhat easier to return to the text and refocus on the material without too many “distractions”.

Step 3: Multiple readings with different emphases

The text underwent multiple readings with different emphases. Initially, a descriptive approach was taken, highlighting and trying to “be close” to the participants’ experiences. The second reading centred on linguistic aspects, encompassing pronouns, keywords, emotion words, pauses, laughter, fluency, and the frequency of concepts. While the first two readings aimed for closeness to the participant’s experiences, the third reading embraced the researcher’s perspective through conceptual insights, fusing more explicitly the informant’s descriptions with the researcher’s interpretations.

Step 4: Identifying, highlighting, and clustering quotes

To keep experiential statements (step 5) grounded and avoid premature abstraction, we introduced a step involving in-depth exploration of quotes from the transcript. In each focus group, 184–256 quotes were selected and emphasised. In the analysis, all quotes were marked with participants’ pseudonyms, so it was possible to follow the specific participant and take an idiographic account. Subsequently, we conducted a level 1 clustering of quotes within each focus group, addressing questions such as theoretical and practical connections, key experiential features, and participant experience and sense making processes.

Step 5: Constructing experiential statements

Then, the clusters of quotes were reviewed and refined, given descriptive names, and constructed into a set of experiential statements [48] (pp. 86–87).

Step 6: Searching for connections across experiential statements in the particular focus group

The next step was to make a level 2 analysis of the experiential statements for each particular focus group by reclustering the level 1 clusters. The new level 2 clusters were given descriptive names when this was complete.

Step 7: Searching for connections across group experiential statements across the focus groups

One of the final steps entailed exploring the interconnections between the focus groups within the scope of the level 2 group experiential statements. As a result of this process, a total of 21 level 3 group experiential statements or themes were derived. Subsequently, the first author was responsible for the last selection and conceptualisation, which was guided by the research question in this study.

Step 8: Checking individual transcripts to ensure nothing missed

After the final grouping and conceptualisation, the first author reviewed all the transcripts to ensure that crucial ideas and quotes were included in the final write-up.

This analysis constructed three principal findings, which will be presented in the results: (1) Is it safe enough? (2) Do we have a joint and regenerative project? and (3) Three key sources for sense making.

## 3. Results

Our IPA analysis led to three main group experiential themes across the four focus groups. These themes appear to capture and shed light on the process through which the couple therapists decide whether or not to continue with conjoint sessions when violence has become an issue. Collectively, these three main group experiential themes constitute what we have termed as “clinical sense making”. The first two emerged as questions and thematised “what” the therapists are oriented towards to determine whether to continue with conjoint sessions when violence has become an issue; and the third group experiential theme highlights “how” the therapists try to make sense of and understand the situation: (1) Is it safe enough? (2) Do we have a joint and regenerative project? (3) Three key sources for sense making.

The three different group experiential themes will be presented separately in an analytical attempt to highlight significant aspects regarding “the what” and “the how” in the “clinical sense making” process of couple therapists deciding whether to continue with conjoint sessions when violence has become an issue. We employ narrative descriptions and figures to emphasise key aspects. Tables indicate the presence of sub-themes in each focus group. Selected quotes from the interviews are used to help the reader substantiate the analysis and the authors’ interpretations and, at the same time, capture the essence of the participants’ sense making and experience about each theme. However, it is essential to note that in clinical practice, these themes are intertwined and simultaneous rather than isolated phenomena. The discussion and clinical implication section will delve more into their interconnectedness and relevance to practice.

### 3.1. Group Experiential Theme 1: Is It Safe Enough?

Throughout the interviews, the therapists positioned themselves and articulated a core assumption that conjoint couple therapy is a potent format for addressing underlying relationship issues, even in the presence of violence, as long it was safe enough. The therapists also addressed how couples often expressed a desire to confront and resolve problems together, resisting the idea of individual therapy. However, in all focus groups, research participants emphasised the primary safety concern when violence became an issue, and it was the first sign of whether continuing with conjoint sessions was appropriate. In particular, the therapists were orientated towards what they perceived as “red flags” linked to the risk for escalation or new violence. Concurrently, they grappled with a sense of uncertainty regarding continuing the collaboration within a conjoint couple therapy context. As articulated by Nora (FG1):

*“I think it’s an uncertainty that I feel, and sometimes I can get anxious about maintaining the same pattern that’s already there… It feels scary to be part of something that maybe makes it even harder for the couple.”*

Many therapists acknowledged that uncertainty remained an inherent aspect of the collaborative process despite their efforts to assess and manage risk and establish safety. This ongoing uncertainty does not negate the importance of these efforts. Instead, it underscores the need for an ongoing iterative process assessing and managing risk and that the focus needs to be on safety throughout and regularly monitored, as Herman (FG2) articulated:

*“Is this safe enough? Maybe, okay yes, this is safe enough, and we have a common goal. However, then, (pause) I think, it may not be after all. Down the line, sort of, that it could change. Um, or the violence, in a sense, can continue in hiding… It’s sort of something you have to, or continue to be a thought then, you must be open to it.”*

**Figure 1:**
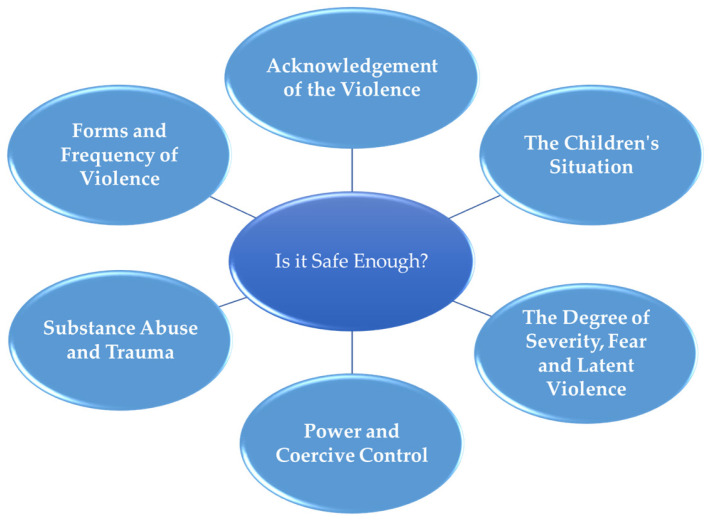
Group experiential theme 1: is it safe enough?

Against this backdrop, what does the therapist focus on to gain greater clarity and confidence around whether or not to continue with conjoint sessions? Through our analysis, we constructed this first group experiential theme out of six foci through which the therapists oriented themselves (see Figure 1 and Table 2).

#### 3.1.1. Forms and Frequency of Violence

The initial point of enquiry for the therapists when violence became an issue revolved around obtaining a comprehensive understanding of the nature and occurrence of violence within the context. A common thread emerged across all four focus groups with participants collectively delving into two critical aspects: the various forms of violence and the frequency of these incidents. Violence can manifest in various ways, encompassing physical, psychological, sexual, material, digital, and financial forms. So, the therapists tried to identify which forms of violence were present or not, along with the frequency of the violence and, thereby, to try to differentiate between isolated incidents and recurring behaviours. Across the focus groups, a consensus emerged, which was particularly highlighted by tone (FG3):

*“If there is severe aggravated violence, repeatedly over time, then we would be much more…, so we probably would not start couple therapy.”*

Stig (FG 1) described and elaborated how he was concerned with the couple’s situation at present, how the violence had developed over time, as well as their thoughts and expectations of how this would develop in the future:

*“How has it been in the past, and what do they think about the future, for each one of them? That can inform different perspectives on how long this has been going on? How has the development been? Has it gotten worse? Has it been static? Has it gotten better? What do you think it will look like in five years? I think these questions often help both me and them to say something about, what shall we do now?”*

In all focus groups, participants acknowledged and underscored that behaviour or frequency, in isolation, may not offer sufficient insights without also considering the relational context in which the violence unfolded. So, creating a formulation with these aspects was crucial to them.

#### 3.1.2. Patterns of Power and Coercive Control

In the focus groups, therapists frequently made a clear distinction between two types of intimate partner violence: coercive controlling violence and situational couple violence. They invoked the term “coercive controlling violence” to characterise instances of violence that unfold within a framework and a “regime” of coercive control, where one partner deliberately employs violence and their power in harmful ways against their partner. In contrast, “situational couple violence” was recognised as a consequence of escalating conflicts in which one or both partners use violence, not out of an intention to control, but because of anger and frustration over a specific situation. As emphasised by Ingvild (FG3), who had just met a couple where she assessed that the violence occurs in the context of “coercive controlling violence”:

*“If you subject someone else to a regime, that becomes very, very, if people start doing things they don’t want to do, or they don’t do things they want to do. And that, in addition, is systematised over time with a certain intensity. I think there’s quite a lot of that.”*

Jacob (FG1) also addressed this:

*“If there is so much fear or it is too unsafe, and the imbalance is so great, I have mostly split them and talked to them individually, then you as a therapist start to think of other ways to go than conjoint couple therapy.”*

As a result of this awareness, the therapists remained sensitive to whether the violence was embedded within a “coercive regime” and, therefore, restricted the possibility of conjoint sessions.

#### 3.1.3. The Degree of Severity, Fear and Latent Violence

The previous quote addressed something the therapists often associated closely with coercive control: the degree of severity, fear and latent violence. They suggested there is not a necessary direct correlation between the actual acts of violence and the consequences for the couple and family because individual, relational, and contextual risk and protective factors may mediate the experience of the violence. However, the therapists emphasised a crucial distinction for them in their decision making as to whether the violence had led one or both individuals in the relationship to adopt maladaptive behaviours in their daily lives, as Thomas (FG2) explained:

*“I have a couple now where there was a violent incident 13 years ago, a very severe incident of violence. And, and it’s a very illustrative example then, where, of how serious violence can be then. Because it still controls their lives really…Um, and it has been a big part of their family life for all these years until now.”*

Nora (FG1) had just started to work with a couple where violence quickly became an issue, and she said:

*“I have a new case like that, where it’s very much latent violence. He says there haven’t been many incidents in quite a while. And she agrees with that, but it is the latent violence that she struggles with, how she navigates to avoid new episodes, and that she’s afraid of it. She walks with that fear within, despite there aren’t many new and concrete episodes.”*

Therapists view the presence of fear and latent violence as indicators of the severity of the violence and its retrospective impact. So, in the context of continued collaboration in a conjoint setting, several therapists saw how clients adapt their relational interactions, and this behaviour was seen as a potential cue for guiding interventions and the couple’s ability to engage in therapeutic work. Therapist were also concerned about whether these relational interactions and behaviours manifested within the therapy room. So, if present, there was little point for them in using the format of conjoint couple therapy because of the fear and lack of safety, which characterised the relationship and influenced the parties in such a way that they could not speak sufficiently freely and thus could not thematise and work with the destructive patterns to enhance safety. As Emma (FG3) put it:

*“In a way, it is a prerequisite to creating a sense of safety that they will be able to talk about something that difficult.”*

#### 3.1.4. The Children’s Situation

Despite the context of couple therapy, all focus groups addressed and emphasised the importance of considering the children’s situation and parents’ caregiving capacity when violence emerges as an issue. This consideration serves a dual informative purpose: if measures are necessary to ensure the children’s safety and development, and the appropriateness of conjoint couple therapy as the intervention at the given time. The participants believed that children exposed to, and witnessing couple violence are at greater risk of developing mental health issues, developmental and behavioural problems. They also recognised that violence within the adult relationship impacts the parents’ caregiving capacity, diverting their attention from the children’s and their own needs. So, in some cases, they considered the family and children’s situation to be too fragile or uncertain to continue conjoint sessions. For example, Ingvild (FG3) had recently worked with a couple where she assessed that the children’s situation was critical and that conjoint sessions and couple therapy were inadequate. She recommended that Child Protective Services (CPS) investigate further due to the distressing circumstances.

*“We have these cases and where we think that here there are quite different weighted power relations in the relationship, here there is a high degree of psychological violence, here there are some children who are quite harmed by it, and then we think: ‘but I can’t do anything here’. Here it is too fragile, here it is too depleted, here they are too worn-out. Then we send a report of concern (CPS), and then we get that dismissal back, where you then think, what do we do now? So, then there are some people who don’t get help, and here are some kids who are suffering. And I’ve assessed that I can’t do anything here because it’s too depleted, too severe, and impossible for the Family Counselling Services to do good enough work here in this case. I think there is someone who, first and foremost, must go in and inquire how the heck those kids are doing! And then it gets dropped!”*

So, therapists frequently encountered dilemmas, discomfort, and emotional strain concerning the children’s well-being, irrespective of their interactions with the children. Consequently, the child’s perspective was prevalent across all focus groups. Nevertheless, there was notable variability in how the therapists described the extent of their direct contact and involvement with the children.

#### 3.1.5. Substance Abuse and Trauma

All therapists highlighted that couples in “violence cases” often struggle with other life stressors and additional challenges that can significantly impact functioning, the risk of recurrent violence, and the potential for engaging in couple therapy. Among these different struggles and complexities, substance abuse and trauma emerged as particularly salient. Several therapists highlighted how trauma responses related to the current partner or previous relationships could create a pervasive sense of insecurity and lack of safety, leading to the belief that conjoint sessions might not be suitable. Two of the therapists worked together with a couple where the female partner was the aggressor in the current relationship, but had a history of severe trauma responses from a prior relationship. Sigrid (FG3) said:

*“Lots of abuse, and you name it. So, so it’s a question of whether she’ll ever come out of going into old feelings all the time.”*

The two therapists thought she was overwhelmed and physiologically flooded, so interactions escalated during the sessions with her new partner, making it difficult to regulate and lead the conversations. Therefore, the therapists decided that individual sessions would provide a safer format. In focus group one, they were also concerned with how substance abuse makes the sessions and therapy project more unmanageable and unpredictable and creates uncertainty, Sunniva (FG1):

*“In some of these cases, substance abuse is also involved, and it is also a complex factor that has to do with safety and insecurity. Sometimes it is the person who is exposed, and sometimes it is the person who commits the violence that has a substance abuse problem.”*

Substance abuse was something the therapists considered to be a heightened risk for new violence and considered it essential to gain “control over” before conjoint sessions and couple therapy could feel safe enough.

#### 3.1.6. Acknowledgement of the Violence

One of the challenges of addressing violent behaviour that the therapists encountered was that it could trigger defensive reactions, such as minimising, trivialising, or rationalising the violence and its consequences. People may also become entrenched in a pattern of blaming others or justifying their actions. Nevertheless, all the focus group participants advocated that it was a prerequisite for even considering conjoint sessions to be appropriate and safe enough i.e., that there was “sufficient” acknowledgement of the violence from both parties. Tone (FG 4) reflected on a case she was co-working with a colleague in the same focus group, where they had decided they could not continue for the moment with conjoint conversations. They experienced and thought that:

*“There is no acknowledgement of the violence, like the guy we worked with, and then we don’t go into it, it becomes like a criterion. We have to see that recognition.”*

Without such acknowledgement, it made no sense to continue with conjoint sessions for the therapists.

### 3.2. Group Experiential Theme 2: Do We Have a Joint and Regenerative Project?

**Figure 2:**
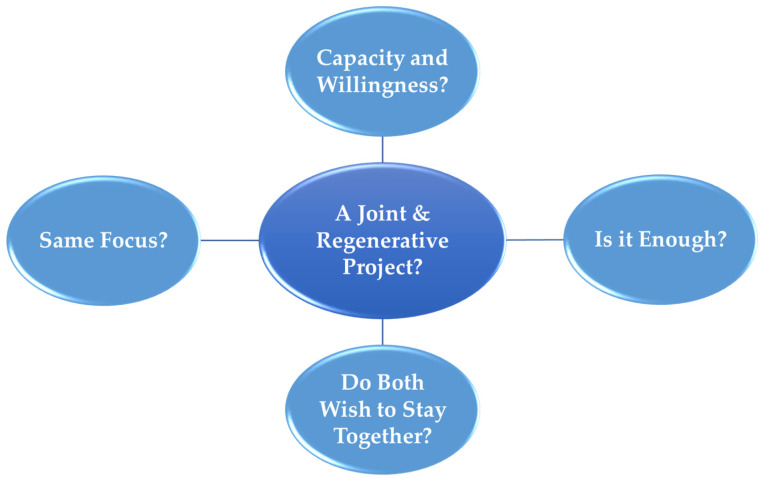
Group experiential theme 2: do we have a joint and regenerative project?

The first group experiential theme was “violence specific” and was oriented toward what the therapists perceived as signs linked to severity, the imminent risk for escalation or new violence, and the question “Can it be safe enough?”. In contrast, the second group experiential theme addressed therapy and cooperation more generally. This group experiential theme incorporated four foci, extracting what the therapists orient themselves towards regarding whether they experience a joint and regenerative project as possible (see Figure 2 and Table 3).

While some therapists emphasised and oriented themselves mainly towards the different aspects found in group experiential theme 1, others focused almost equally much on the experience of having a joint and regenerative project when deciding if conjoint couple sessions were or could be constructive. The following extensive quote from Ingvild (FG 3) summarises many of sub-themes of the second group experiential theme that will be deconstructed and further elaborated below:

*“In my experience, it is, in a way, not how severe the violence is that decides. I’ve had quite a few cases and have now, where there’s been a stranglehold, physically damaging violence, and visits to the emergency room. So that’s not what necessarily excludes me from thinking that we can do something together. It is the extent to which both parties are willing to take responsibility, look at their contributions and are, in a way, prepared to work seriously and prioritise from session to session. So it’s like, yes, it’s people’s motivation, ability, and willingness to look at what they’re doing, which is important in the first place. But then there’s something about when we experience that it doesn’t have the progression we hope for. In terms of frightening events coming to an end, um, what are we doing then?”*

#### 3.2.1. Do Both Partners Wish to Stay Together?

Many therapists thought it would be vital to ask questions regarding the premise that is most often the basis for the couple making contact with the NFCS, namely that they want to strengthen and maintain their relationship. So, do both partners wish to stay together? Nora refers below to a recent experience:

*“In the first session, he’s very keen for it to be a couple therapy, and find back to each other or repair and strengthen the relationship, while she leans more out of the relationship.”*

Many therapists have had similar experiences, whether it was in the start-up phase or it became more apparent as the process and collaboration developed. Nonetheless, several believed that it would be inappropriate to work with them in conjoint sessions if it turned out that one wanted out of the relationship. However, most focus group participants acknowledged the rarity of such situations being entirely clear-cut and that there was often great ambivalence for the couple regarding whether they wanted to invest in and try to “save” the relationship. Several therapists experienced and saw this as an opportune moment for couples to explore and safely discern their path forward preferably with the therapist’s guidance rather than trying to navigate this process alone. Stig, from the first focus group, reflected on this:

*“I think there are a striking number of cases in that area of clarification. So it’s nice then, I think, that those people get the opportunity to reflect with themselves and their partner on what they shall do now. Before we move on, so they don’t rush into either strengthening or breaking up the relationship.”*

Petter (FG 2) had comparable experiences; however, he also emphasised that in several couples he encountered, it seemed that one individual in the relationship initiated conjoint therapy because they needed a safe environment to discuss and commence the process of ending the relationship:

*“Yes, and many of those who come to us in a clarification phase, often one of them have decided in advance that they are not going to continue with this, but need a safe process on it.”*

So, a crucial point of clarification is whether the couple wishes to remain together. However, as evident from the quotes above, there can be feelings of ambivalence or a significant likelihood of a break-up. Nevertheless, the therapist might find it meaningful to continue with conjoint sessions. Their intent is not necessarily to “bring the couple together”, but rather, they believe that conjoint sessions could aid the couple in finding a safer path forward.

#### 3.2.2. Same Focus?

The therapists also play a crucial role within the “therapeutic system”; this aspect was a recognised theme across all the focus groups. Therapists emphasised the significance of alignment and shared focus, stressing that both the therapist and the couple need to be committed to the therapeutic project irrespective of the specific goals they aim to achieve. Nora (FG 1) encapsulated this idea when she remarked:

*“It’s a challenge that sometimes I think, oh my God, you have to break up, long before the people in question think so, and then it’s not certain that I am a very good therapist, if I have a different solution in my head, than they do themselves. So it’s tough, like that tipping point, how am I going to be able to follow somehow closely enough on the client’s process then?”*

The notion of the “tipping point” was a recurring topic across all the focus groups, referring to the juncture at which the therapist had difficulty continuing with the therapeutic project of one or both individuals within the couple. Within this context, it became apparent that certain therapists displayed a higher degree of self-assuredness in “stepping forward” and making these decisions. In contrast, others struggled with an internal conflict around departing from their established therapeutic practices and principles. A significant discussion unfolded in focus group two, initiated by Thomas, who recounted a case in which he had expressed to the couple:

Thomas: *“I don’t think you should be together, right? To be so clear, I think that’s hard. Because it goes so against what we’re drilled on over the years, in a way.”*

Herman: *“Yes.”*

Thomas: *“So to take a, um, such a position then, I think that’s difficult.”*

Herman and Petter: *“Mmm, yes” (agrees).*

Thomas: *“And that, and then I feel it straining in me, when I did it. And it’s kind of puzzling then, because it’s not, it’s not those people really, it’s, it’s the culture in a way I’m embedded in and that I’m stepping out of, in a way, that we shouldn’t do that.”*

Despite different degrees of self-assuredness, they all acknowledged the necessity of adopting a distinct approach when dealing with issues of violence compared to cases that lacked such dynamics, challenges and potential concerns for safety.

#### 3.2.3. Capacity and Willingness to go to therapy?

A vital aspect concerning the viability of a joint and regenerative project revolved around the therapist’s evaluation of the couple’s capacity and willingness to actively participate in the processes required for behavioural and situational change. Ingvild (FG 3) elaborated on this concept when she stated:

*“Thank goodness we are able to change behaviour, we humans, but that requires an enormous presence and capacity that is quite fierce.”*

This notion of capacity and willingness encompassed various dimensions. On the one hand, it entailed considering the socio-economic and situational conditions affecting the couple. On the other hand, it delved into the therapist’s perception of the client’s individual’s current dispositions and attitudes towards actively engaging in the processes to mitigate the risk of further violence and enhance safety for all family members. Ingvild and Sigrid (FG 3) elaborated and offered insights into this dual perspective:

Ingvild: *“So I’m trying to get both capacity and motivation. And then at the same time willingness and even ability…and we really shouldn’t overestimate our importance. To think that people who are so “genuinely struggling in life”; there’s Covid and they are from other countries, they haven’t had contact with their family of origin for a couple of years, they’ve had a child along the way, right. It’s not their language, they don’t necessarily understand anything particularly about the Social Security System, that doesn’t mean the heck I do either. And it’s, well, they’re so depleted and struggling, aren’t they…”*

Sigrid: *Mmm (agrees).*

Ingvild: *“…they are so exhausted, to meet them and think that with an hour here every two weeks we will make a big difference, it’s a bit naive.”*

Hence, in some instances, the decisions regarding whether to continue or discontinue conjoint sessions were grounded in recognition of the couple’s incapacity, stemming from contextual factors rooted in a systemic perspective. Other times, there were situations where the therapist’s perception of the couple’s commitment and motivation to effect change in their current situation influenced the decision. Jacob, a participant from focus group one, shared:

*“If you’re satisfied in your “current life” or have too much control of the situation. Either way, it’s difficult, through talking, getting them to think: oh, now I really have to pull myself together because this isn’t good for us. That’s not easy.”*

Simultaneously, therapists underscored that the concepts of capacity and willingness should not be viewed as binary attributes where they are either entirely present or absent. Instead, they advocated for a more nuanced perspective, considering them as variables that exist along a spectrum, ranging from robust to restricted. They observed that these capacities could fluctuate over time and could also be a primary focus of attention and intervention aimed at bolstering the couple’s ability and motivation to reduce the risk of violence and enhance safety. Nevertheless, therapists stressed that even at the initial stage, a minimum level of capacity and willingness must be evident to make conjoint sessions a feasible option.

#### 3.2.4. Is It Enough?

The couple’s capacity and willingness to establish a safer living environment for all involved is undoubtedly crucial. However, therapists also underscored their responsibility in evaluating whether the collaboration and what they, as therapists and the NFCS, could contribute was enough and/or if there were other services more suited to meet the needs of this family. This was essential immediately after the violence became the primary concern but also needed continuous monitoring. Sigrid from focus group two said:

*“And then there’s always that consideration of whether there’s better services elsewhere.”*

Her colleague Ingvild referred to a conversation in which she had discussed with the couple that the services from the NFCS alone would not be sufficient as she had assessed it and that it was essential to be transparent and discuss this with the couple:

*“And I said that to the couple. That I think this is not fair to you. No, because it’s too insufficient help at this point, so how can we think about the situation then?”*

The therapists in focus group two had too often experienced discomfort in that the resources and framework of their family counselling office degraded the services they initially could offer and believed the couple needed in order to create regenerative processes. Their primary concern revolved around the challenges of scheduling regular and frequent sessions and, if required, involving a co-therapist in the process. Sigrid and Ingvild (FG 3) articulated their experience, noting that when violence entered the equation, their immediate instinct was to arrange follow-up appointments promptly, but that was complicated and rarely possible and, therefore, created disquiet:

Sigrid: *“Sometimes, it can be challenging to work on these cases, and I think I can’t do it. I have a new free appointment in 6 weeks!”*

Ingvild: *“It’s absolutely crisis.”*

Sigrid: *“And if I’m going to have a co-therapist in, then at least eight weeks pass before I can meet the couple again…And that’s, what we can get very, very exhausted by.”*

In contrast, on other occasions, the therapists felt and believed they possessed the necessary resources and a conducive framework that allowed them to establish regenerative processes and feel a sense of mastery in their work. However, the question whether “it is enough?” remained pertinent. An integral part of this question involved monitoring for any violent incidents between sessions. For instance, Sunniva (FG3) mentioned:

*“It’s been two weeks since we last saw each other, right? Has it been a particularly frightening or particularly scary episode since the last time? How have you experienced it? So, so like that…, I try to get to the worst thing that’s happened since the last time, and then we unpack it behaviourally.”*

At the same time, they were concerned about how the interaction within the couple/family developed over time. Ingvild continued:

*“So it’s both about, does it look like they’re starting to think differently about themselves and their situation? Does it look like this change in perception transfers into the action they actually do? That’s on the one hand, but also to what extent is there in a way any change in meaningful understanding of what is happening to them, and what they themselves contribute, are they helping or maintaining the situation?”*

So, in this context, the therapists underscored the importance of ongoing evaluation to determine whether the collaboration was progressing in the right direction and if the measures were enough or if other actions were needed.

### 3.3. Group Experiential Theme 3: Three Key Sources for Sense Making

**Figure 3:**
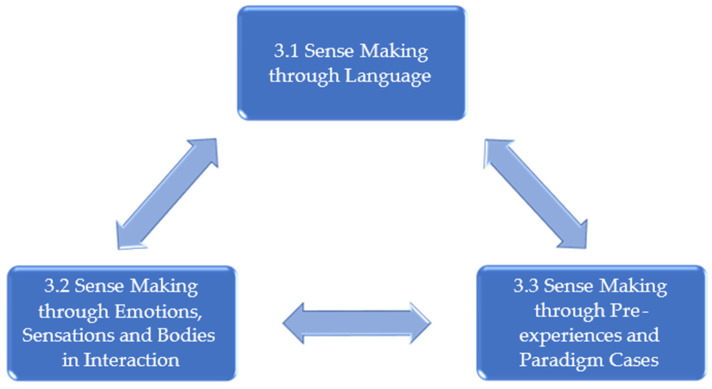
Group experiential theme 3: three key sources for sense making.

The third group experiential theme describes three key sources regarding how the therapists “read” the situation and how the therapists informed their decisions. The therapists used three key sources to make sense of the two questions that constituted the two preceding group experiential themes: “Is it safe enough?” and “Do we have a joint and regenerative project?” The three sources were: Sense Making through Language, Sense Making through Emotions, Sensations and Bodies in Interaction, and Sense Making through Pre-experiences and Paradigm cases. There is undoubtedly overlap among the three sense making resources, and in the analysis, they will inform each other. So, it is not that the therapists inform their choices from one or the other resource, it is more of a matter of focus and priority, which modality the therapist can access and, if applicable, chooses to prioritise at any given moment (see Figure 3 and Table 4).

#### 3.3.1. Sense Making through Language

When it comes to determining whether to continue with conjoint sessions, it seems therapists heavily rely on the process of sense making through language. Language is not only a medium through which therapists reflect upon and express their thoughts and reflections regarding their decisions in this study, but language, in general, also serves as the primary source that guides the decision making process in which they are embedded.

In addition, and more specifically, therapists employed various theoretical or practice-based terms and concepts to understand and initiate inquiries and assess different aspects that they consider relevant and significant for their decision. The subordinate themes within group experiential themes 1 and 2 provide examples of these terms and concepts actively incorporated by therapists. These terms and concepts were potent “tools” and sources that brought forth and allowed certain aspects to appear in the “light”.

One concrete and illustrative example is the concept of “power and coercive control”. Despite slight variations in terminology and wording among the therapists, all 12 consistently referred to this concept throughout the focus group interviews. While providing precise operational definitions is possible, the therapists did not consider precise definitions as the primary or foundational aspect of their decision making. Instead, they used it more loosely as a “perceptual lens”, guiding their inquiry and exploration without providing a rigid blueprint of what to seek. It seemed they used the concept to draw their attention to essential features they thought would provide a relevant starting point for inquiring and building a sufficient foundation for their sense and decision making. For instance, in focus group two, Thomas frequently used the “power and coercive control” concept as a point of entry and a framework for examining various facets of the couple’s daily life.

*“I thematise equality and power and coercive control in the relationship. How it feels, um, and that’s a way for me to check out, my concern then. If I get, get more worried, after checking out that topic, um, in relation to, who makes decisions, relative to, it could be about finances. It could be about, about, that with respect to each other: Um, and that’s a way of checking out, was my hunch right?”*

Sunniva (FG3) also used power and coercive control as a term and concept when violence became an issue and a “perceptual lens” to assess whether the conjoint sessions were appropriate to proceed with:

*“To go further into what, what else is there in that relationship of different power aspects then, i.e., what kind of powerlessness is it that who experiences, in what situations, and what kind of power positions exist, what does that do to the dynamics of the relationship and communication then?”*

These concepts clearly contributed to describing and illuminating different aspects that might otherwise have been overlooked or lost by overwhelming information and impressions.

#### 3.3.2. Sense Making through Emotions, Sensations and Bodies in Interaction

Several therapists employed terminology such as “mood in the room”, “vibe”, “I had a hunch”, and “I sensed”, or “intense feelings and atmosphere” when describing their intuitive perceptions of the ongoing dynamics within the therapeutic context.

So, the second primary source of information for the therapists in how they “read the room” were their intuitive perceptions of the ongoing dynamics within the therapeutic context, more specifically, their experience-based embodied feelings and sensations and their impression of the couple’s bodies and interaction in the room. They regarded this source of information as indispensable to making sense of the intricacies of the conjoint sessions, guiding their interventions, monitoring the therapeutic process and informing assessment and management of risk. While all four focus groups addressed this aspect to some extent, the discussions in focus groups 1 and 4 delved more deeply into the therapists’ sensitivity to and active interpretation of these non-verbal dynamics, as illustrated by Stig from focus group 1:

*“That dynamic, coming in from the waiting room, as they walk into the office, how they sit down, how they talk, don’t talk to each other, also how they, too, how they react when I say that I want to have individual conversations with them, that is, all these responses then. Both how they manoeuvre in relation to each other and in relation to me, it gives a lot.”*

Experiencing these interactions makes bodily impressions, and the therapists’ sense making of the interaction in the room helps the therapists to consider which measures and alternatives of action can be seen as feasible. Tone elaborated (FG 4):

*“If it’s very, if there’s a feeling or atmosphere so intense as to seem almost tangible in the room, one that’s very tense, trembling, one of them don’t make eye contact, and the other is very, what should I say, one is very eager to speak, and lays out the situation. Then I can feel the discomfort and distress that, here’s something, um, and it kind of stops the possibility to go as far into the landscape as I might have.”*

When the therapists experienced this bodily discomfort and became uncertain about how it was most appropriate to proceed, several participants described this as an inner dialogue and said that they did make intuitive decisions that they believed best safeguarded safety. This internal and intuitive decision making process posed a dilemma for some, as it contradicted their aspiration for openness and transparency within the conjoint sessions with the couple. Thomas, who participated in focus group two, offered reflective insights into this intricate balancing act:

*“I don’t sit with that sensation without doing something then, so I’m kind of like, I’m always doing something when I get a hunch like that, or, if talked about, it’s rare, so I try to be transparent then, so, so I’m probably transparent when I’m working with couples… But that has to be my assessment of, what can I say, or how worried am I that something wrong will happen then? Yes, if I am sufficiently afraid of it, I will contact my colleague or manager. Um, or, or am I sufficient, then I will, in a way, wait to talk about these things and check it more out in individual conversations.”*

Some described this sensitivity and capacity to register and interpret one’s own and other’s bodies, emotions, and interactions as indispensable in deciding the appropriateness of conjoint sessions. At the same time, several therapists described this process of interpretation as challenging to put into words, rather “operating” as a sensation influencing their impression and action.

#### 3.3.3. Sense Making through Pre-Experiences and Paradigm Cases

The study participants consisted almost exclusively of experienced therapists with long tenure in the NFCS and more than average experience working in cases where violence was one of the main concerns. It would be unlikely to claim that their existing ideas and prior experiences did not influence their sense making and decisions. These therapists carried a weight of past experiences, particularly some paradigm cases that had left a lasting impression on their awareness and bodies. They vividly recounted how these experiences came to the forefront and influenced their “clinical sense making” in new and similar situations, bringing forth more or less apt suggestions for decision making and action. Stig, in focus group 1, talked about how his experiences from several cases have accumulated and contributed to him becoming more alert and led to a more significant curiosity and inquiring than he did previously:

*“I’ve probably been scared myself, when I’ve discovered afterwards how ugly it has been and how close to death one of the parties has been, so it’s probably made me even more, maybe sensitive, but very alert to asking what kind of violence are we talking about? How far does it go? How hard do you hit? What did you use? Right? How serious is this in terms of survival? Because I’ve become, maybe I’ve been secondarily traumatised (laughs), but I’ve been a little intimidated a few times by how close it’s been in terms of going awfully wrong.”*

At other times, it was not multiple and recurrent experiences that were forming the therapist’s practice and “cognitive mind maps” related to their decision making, but rather, it was a singular event or a paradigm case that had a lasting influence. Towards the conclusion of the fourth interview session, as the participants were invited to share any additional insights, Emma, a seasoned therapist among the focus group members, recounted a case from her past that dated back almost a quarter of a century. This particular case had etched a profound and enduring impression on her, as she said:

*“I had a couple where I felt very strongly there was something wrong. He was quite big, and she was small, and she was very tense. I had a couple of conversations with them, and then lost them. And I thought, there was probably violence there, I’ve thought subsequently. And it’s comes to me, many times since then. I have that picture with me. They slipped through my hands. At that time, I didn’t have enough training and was too new, but that picture I have, of that tension and anxiousness. So I can recognise it. Right, I see, very. It’s stuck like such a powerful memory. When I see it, in a way, I recognise that tension (in new cases)… It affects me, now I need to be more sharpened.”*

Some of the therapists described how their pre-experiences and their paradigm cases shaped their sensitivity to specific aspects and foundational understanding. So, in some instances, the weight of past experiences was experienced as a resource, and other times, it was a constraint. Nonetheless, it provides a framework for evaluating the information and complexities when assessing the new situations of the cases they encountered.

## 4. Discussion

**Figure 4:**
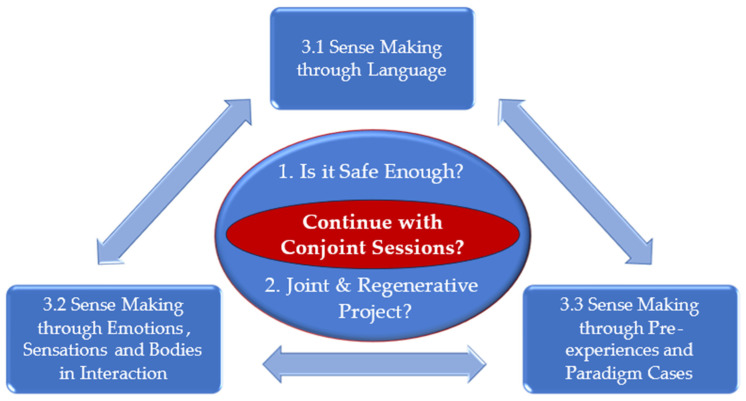
Visualising the interconnection of group experiential themes 1, 2 and 3 with the main research question.

Partner violence presents a significant challenge in the realm of couple therapy. This article has explored “clinical sense making” among couple therapists as they grapple with and try to navigate whether to continue conjoint sessions in the presence of violence. The article offers unique insights regarding “what” the therapists orient themselves towards and “how” they try to form an impression of whether to continue with conjoint sessions. The therapists’ descriptions of “clinical sense making” reveal that this process is not confined to identifying the mere presence or absence of violence; rather, it delves into the unique context of each case, characterised by intuition, holistic consideration, and inherent complexity and uncertainty. It also highlights therapists’ processes and dilemmas when committing to safety and pursuing regenerative processes. Simultaneously, they recognise the limitations of conjoint sessions and that not every couple may benefit from them (see Figure 4).

After violence has become an issue in couple therapy, the first stage of clinical sense making is to form a first impression of the situation for the next steps in the process. Even though this is often the phase where the therapist has the least information, the “raw data” can still be comprehensive and overwhelming. Schön describes that the problems we face in professional practice are not necessarily clear and straightforward. We often stand in situations that are characterised by uncertainty, instability, and conflicts of values. He writes: “The problems of real-world practice do not present themselves to practitioners as well-formed structures. Indeed, they tend not to present themselves as problems at all but as messy, indeterminate situations” [59] (p. 4). When the world appears chaotic and unruly, individuals themselves must create meaning through their constructions and definitions. This distinction making process is an interpretative and sense making act [60]. When we construct and make sense of a problem, we do this through a: “…complementary process of naming and framing. Things are selected for attention and named in such a way as to fit the frame constructed for the situation” [61] (p. 146). So, when therapists encounter this complex or ambiguous situation when violence has become the primary concern, they instinctively seek to reduce uncertainty and make the world more predictable. The analysis from this study shows that the therapists try to make sense by making distinctions, i.e., by “naming” and “framing” through the “what’s” (group experiential themes 1 and 2—is it safe enough; and do we have a joint and regenerative project?). This process shapes the therapists’ interpretation and creates a “lens” that influences and gives an impression of a situation.

This lens through which the therapists interpret the world around them depends on the individual’s prior knowledge, beliefs, values, and past experiences. From Gadamer’s perspective, prejudice is not a hindrance but a positive prerequisite for understanding something at all [62]. So, without our historically determined prejudices and horizons of understanding, there is nothing to draw upon to make sense of the situation. So, attempting to understand and make sense of a situation will always involve placing the phenomenon of understanding in the context of our pre-understanding and prejudices, which are always related to a particular historical and social context.

Through group experiential theme 3, we present “how” the therapists try to make sense and form an impression of the situation by drawing upon three distinctive sources of information: “Language”, “Emotions, Sensations and Bodies in Interaction”, and “Pre-experiences and Paradigm Cases”. We have used the term “impression” in several places throughout the text. Sara Ahmed [63] suggested that using the term “impression” can constructively blur the boundaries between bodily sensations, emotions, cognitive processes, and evaluation. This can serve and allow us to transcend rigid analytical distinctions between these components of human experience and, at the same time, acknowledge the interconnectedness of these domains. So, using the term “impression” in the context of how therapists make sense in this article relates to the interplay between perception, cognition, sensation, and emotions and how these domains impact our impressions. Once the therapists have formed a comprehensible impression of a situation regarding safety and assessed the viability of a joint and regenerative project, this impression serves as a springboard and foundation for clinical considerations, decisions and action regarding these matters.

There is a constant balancing act throughout the process of making sense of the situation and deciding whether or not to continue with conjoint sessions. One of the main aspects of this act is balancing ethical considerations. These are both implicit and explicit in all focus groups: a commitment to go beyond merely identifying and taking a moral positioning against the violence but also to contribute to enhanced safety and healthy relationships [64]. Simultaneously, they elucidate the delicate balance they must strike between their dedication to fostering generative processes and their ethical and professional obligation to safeguard the well-being of each individual in the couple and their children.

The therapists operate within a broader organisational and legal framework; however, it is noteworthy that in this study, they seldom explicitly reference these formal structures when describing and discussing how they navigate, make sense of, and decide whether to proceed with conjoint sessions. This may suggest that the therapists, either independently or in conjunction with their colleagues, primarily shoulder the responsibility and facilitate these decisions independently with limited support from these established frameworks. This could be one piece of the puzzle as to why therapists often report a profound sense of burden and personal responsibility in dealing with such cases. On the other hand, all focus group participants emphasise the necessity of maintaining a continual and dedicated professional focus on addressing violence within the organisation and at the individual family counselling office level. This commitment is vital as it plays a significant role in enhancing individual therapists’ capacity to excel in their position to offer safe and tailored services, thereby optimising the possibility of making a meaningful contribution when dealing with these cases.

### 4.1. Implications for Therapeutic Practice

Building individual capacity: In dealing with partner violence cases, couple and family therapists must equip themselves with the necessary knowledge, skills, and resources. This involves the ability to address potential violence, even if it is not initially part of the referral, and to assess and manage safety when couple violence arises. Developing a nuanced “clinical sense making” of the complexities surrounding partner violence enhances therapists’ capabilities to navigate, comprehend, and make informed decisions. This requires a reflective therapeutic practice supported by supervision and infused with reflexivity, heightened awareness, and a critical application of knowledge [59,65]. Such critical thinking delves into the “depth” and “breadth” of explicit and implicit knowledge that therapists use in individual cases [66]. Filtering the results from this study through a critical thinking perspective, we wish to forefront and outline three key questions for therapists to reflect on in their daily practice when confronted with partner violence, thereby augmenting their “clinical sense making” capacity in such cases. They are: (1) What do I, as a therapist, orient myself towards to create a sufficient impression to make professional and ethical choices regarding both safety and whether we have a joint and regenerative project? and (2) How do I orient myself and on what sources are my impressions constructed? To respond to this question constructively, the therapist must at least have fundamental “violence-specific knowledge and skills” besides the “ordinary” therapeutic repertoire of navigating more than one client in the room. To enhance the critical perspective within these reflections, the introduction of a third question becomes important for both practice and supervision: (3) What aspects am I overlooking or failing to orient myself towards, and are there alternative sources that could contribute to forming a more comprehensive impression for making professional and ethical choices? However, the capacity for reflective practice and “clinical sense making” deployed by therapists is intricately linked to the organisation’s resources and supportive framework. The therapist cannot shoulder this responsibility alone.

Building organisational capacity: It is pivotal to recognise that the organisation must ensure and facilitate the provision of adequate resources and a supportive framework. This implies a commitment to foster an environment that empowers therapists by allowing and scaffolding: (1) Self-Reflection: Encourage therapists to engage in continuous self-reflection. They should explore their personal beliefs, values, and biases related to partner violence in general and within their specific cases. (2) Supervision: Provide therapists with regular supervision sessions to discuss their cases, including the three questions above and their emotional reactions working with these issues. (3) Training and Education: Offer education and training in couple and family violence, trauma-informed care, including understanding power dynamics and control issues and how this more generic knowledge can be utilised in a couple and family therapy context. (4) Establish routines and agreements for and enable the individual therapist to collaborate with other local agencies and organisations. This interdisciplinary and interservice approach can enrich and provide families with the best services possible.

### 4.2. Reflections on the Limitations and Strengths of the Study and Future Research

We recruited and used existing professional practice groups because we thought that it would be helpful to provide safety, thus enabling more in-depth exploration of a shared and potentially sensitive topic and experiences [48]. We also addressed, at the beginning and the end of each focus group, if any aspects hindered or made it difficult for the participants to share and discuss openly since both interviewers were part of the same organisation. In all the focus groups, they dismissed this and added that they experienced the opposite. Since the researchers were from the same organisation, it made it easier to highlight concrete examples and go “straight to the point”. It also created a more extensive and safer “reflection space” that made it easier to bring up complex, challenging and vulnerable topics. Although the accuracy of such statements cannot be tested, there was little evidence of self-censoring or that these were not valid descriptions.

The recruitment was conducted through purposive sampling. Therefore, the study was conducted with a small and selected group out of the total number of therapists in the Norwegian Family Counselling Services. The represented therapists are likely to have more knowledge, experience and more extensive and dedicated interest in working with couple and family cases where violence is a concern. So, the findings must be seen within that context. However, violence becoming an issue in conjoint sessions is a relatively common phenomenon in different services; therefore, the results may be transferable, in some sense, after filtering through the unique service, social, cultural, and legal contexts.

An essential part of qualitative research and an IPA approach is for the researchers to have a reflective and transparent position through the process [48,58,67]. Therefore, as mentioned above, our “horizon” of understanding, experiences, language, values and context that we are embedded in will be the limitations and prerequisites for the findings and this article as a whole.

Regarding our personal standpoints, they are in different ways, inevitably impacting the research project from the initial phase to the write-up. As researchers and couple and family therapists, we believe and will always strive to prioritise and have the safety and well-being of the individuals involved as the highest context marker for our work. At the same time, we believe that a systemic perspective is a valuable framework for understanding and intervening in cases of partner violence because it promotes a nuanced, contextual, and interconnected understanding of the issue. This does not mean that we believe that conjoint sessions or couple therapy are always appropriate. We argue that violence in intimate relationships is not a uniform phenomenon but is better conceptualised along a multifactored continuum differentiated by different aspects and that these variations must be reflected both in research and clinical practice [21,22,23]. In future research, gaining deeper insights into the experiences of both clients and therapists involved in specific cases can provide a more comprehensive understanding of the subject matter. Furthermore, providing a more in-depth exploration of various aspects highlighted in this article and establishing a closer connection to clinical practice could enhance contributions to both the practice and research fields.

## 5. Conclusions

Throughout this article, “clinical sense making” emerges as an iterative and continuous process crucial for therapists dealing with complex situations, such as partner violence in couple therapy. The article offers unique insights regarding “what” the therapists orient themselves towards and “how” they try to form an impression of whether to continue with conjoint sessions. This dynamic process involves constantly gathering, framing and naming circumstances and information to construct coherent impressions of a given situation. These impressions, in turn, serve as a foundation for subsequent professional and ethical considerations, guiding therapists in making informed decisions and taking appropriate actions. “Clinical sense making” based on this article’s findings can be condensed and defined as “an iterative and ongoing process of gathering, framing and naming circumstances and information into comprehensible impressions of a situation that serve as a foundation for professional and ethical considerations, decisions and action”. However, this process of “clinical sense making” in couple violence cases is far from straightforward. It is characterised by complexity, intuition, and an unwavering commitment to safety and regenerative processes. Therapists recognise the limitations of conjoint sessions and the need for a nuanced, context-specific approach. In navigating this intricate terrain, therapists leverage their blend of formal knowledge and intuitive insights, striving to provide the best possible service to their clients while fostering a transformative path towards safety and healing. Therefore, “clinical sense making” is not merely an academic exercise but a professional imperative that can shape the course of therapeutic intervention and people’s lives.

## Figures and Tables

**Table 1:** Participant sample demographics.

Participant Demographics	Number of Participants
Gender	
Female	6
Male	6
Age	
41–50	3
51–60	8
61–	1
Years in the NFCS	
0–5	2
6–10	5
11–20	3
20–	2

**Table 2:** Presence of sub-themes for Group experiential theme 1, by focus group.

Name of the Sub-Themes	FG 1	FG 2	FG 3	FG 4
Forms and frequency of violence	X	X	X	X
Power and coercive control	X	X	X	X
The degree of severity, fear and latent violence	X	X	X	X
The children’s situation	X	X	X	X
Substance abuse and trauma	X	X	X	X
Acknowledgement of the violence	X	X	X	X

**Table 3:** Presence of sub-themes for Group experiential theme 2, by focus group.

Name of the Sub-Themes	FG1	FG 2	FG 3	FG 4
Do both partners wish to stay together?	X		X	X
Same focus?	X	X	X	X
Capacity and willingness to go to therapy	X	X		X
Is it enough?	X	X	X	X

**Table 4:** Presence of sub-themes for Group experiential theme 3, by focus group.

Name of the Sub-Themes	FG 1	FG 2	FG 3	FG 4
Sense Making through Language	X	X	X	X
Sense Making through Emotions, Sensations and Bodies in Interaction	X	X	X	X
Sense Making through Pre-experiences and Paradigm Cases	X	X	X	X

## Data Availability

More information about the data presented in this study is available on request from the corresponding author. The data are not publicly available due to privacy and ethical reasons.

## References

[1] Visser M., Van Lawick J., Stith S.M., Spencer C. (2020). Violence in families: Systemic practice and research. Systemic Research in Individual, Couple, and Family Therapy and Counseling.

[2] Andersson T., Heimer G., Lucas S. (2015). Violence and Health in Sweden: A National Prevalence Study on Exposure to Violence among Women and Men and Its Association to Health.

[3] Haaland T., Clausen S.-E., Schei B. (2005). Vold i Parforhold-Ulike Perspektiver: Resultater fra den Første Landsdekkende Undersøkelsen i Norge.

[4] Smith S.G., Basile K.C., Gilbert L.K., Merrick M.T., Patel N., Walling M., Jain A. (2017). National Intimate Partner and Sexual Violence Survey (NISVS): 2010–2012 State Report.

[5] Thoresen S., Hjemdal O.K. (2014). Vold og Voldtekt i Norge. En Nasjonal Forekomststudie Av Vold I Et Livsløpsperspektiv Rapport.

[6] Jose A., O’Leary K.D. (2009). Prevalence of partner aggression in representative and clinic samples. Psychological and Physical Aggression in Couples: Causes and Interventions.

[7] Simpson L.E., Doss B.D., Wheeler J., Christensen A. (2007). Relationship violence among couples seeking therapy: Common couple violence or battering?. J. Marital Fam. Ther..

[8] Dahl S., Følling L., Gulbrandsen O. (1983). Registrering av fysisk vold i familier: En undersøkelse foretatt ved familierådgivningskontorene i Oslo. Fokus På Fam..

[9] Flåm A.M., Handegård B.H. (2015). Where is the child in family therapy service after family violence? A study from the Norwegian family protection service. Contemp. Fam. Ther..

[10] Vatnar S.K.B. (2000). Familievold og familievern. Presentasjon og drøfting av en kartleggingsundersøkelse ved Familievernkontorene i Norge. Fokus På Fam..

[11] Stith S.M., Rosen K.H., McCollum E.E. (2003). Effectiveness of couples treatment for spouse abuse. J. Marital Fam. Ther..

[12] Stith S.M., McCollum E.E., Amanor-Boadu Y., Smith D. (2012). Systemic perspectives on intimate partner violence treatment. J. Marital Fam. Ther..

[13] Karakurt G., Whiting K., van Esch C., Bolen S.D., Calabrese J.R. (2016). Couples Therapy for Intimate Partner Violence: A Systematic Review and Meta-Analysis. J. Marital Fam. Ther..

[14] Vetere A., Cooper J. (2001). Working systemically with family violence: Risk, responsibility and collaboration. J. Fam. Ther..

[15] Cooper J., Vetere A. (2008). Domestic Violence and Family Safety: A Systemic Approach to Working with Violence in Families.

[16] Henderson A.J., Bartholomew K., Trinke S.J., Kwong M.J. (2005). When loving means hurting: An exploration of attachment and intimate abuse in a community sample. J. Fam. Violence.

[17] Slootmaeckers J., Migerode L. (2018). Fighting for connection: Patterns of intimate partner violence. J. Couple Relatsh. Ther..

[18] Slootmaeckers J., Migerode L. (2020). EFT and Intimate Partner Violence: A Roadmap to De-escalating Violent Patterns. Fam. Process.

[19] Vetere A. (2023). Working Systemically with Family Violence and Attachment Dilemmas. Attachment Narrative Therapy: Applications and Developments.

[20] Abbott J., Johnson R., Koziol-McLain J., Lowenstein S.R. (1995). Domestic violence against women. Incidence and prevalence in an emergency department population. JAMA.

[21] Carlson R.G., Dayle Jones K. (2010). Continuum of conflict and control: A conceptualization of intimate partner violence typologies. Fam. J..

[22] Dokkedahl S.B., Elklit A. (2018). Undersøgelse af Indbyrdes Vold.

[23] Johnson M.P., Leone J.M., Xu Y. (2014). Intimate terrorism and situational couple violence in general surveys: Ex-spouses required. Violence Against Women.

[24] Stith S.M., Spencer C. (2023). Commentary: 25 Years After Johnson’s Typology of Intimate Partner Violence the Impact of Johnson’s Typology on Clinical Work. J. Fam. Violence.

[25] Kelly J.B., Johnson M.P. (2008). Differentiation among types of intimate partner violence: Research update and implications for interventions. Fam. Court Rev..

[26] Johnson M.P. (2008). A Typology of Domestic Violence: Intimate Terrorism, Violent Resistance, and Situational Couple Violence.

[27] Johnson M.P. (2017). A personal social history of a typology of intimate partner violence. J. Fam. Theory Rev..

[28] George R. (2007). Typologies of intimate violence and assessment: Making the distinction. Fam. Ther. Mag..

[29] Holtzworth-Munroe A., Stuart G.L. (1994). Typologies of male batterers: Three subtypes and the differences among them. Psychol. Bull..

[30] Johnson M.P. (1995). Patriarchal Terrorism and Common Couple Violence: Two Forms of Violence against Women. J. Marriage Fam..

[31] Gottlieb M.C., Lasser J., Simpson G.L., Gurman A.S. (2008). Legal and ethical issues in couple therapy. Clinical Handbook of Couple Therapy.

[32] Aldarondo E., Straus M.A. (1994). Screening for physical violence in couple therapy: Methodological, practical, and ethical considerations. Fam. Process.

[33] McLaughlin K.D. (2017). Ethical considerations for clinicians treating victims and perpetrators of intimate partner violence. Ethics Behav..

[34] Greene K., Bogo M. (2002). The different faces of intimate violence: Implications for assessment and treatment. J. Marital Fam. Ther..

[35] Vetere A. (2006). Commentary-The role of formulation in psychotherapy practice. J. Fam. Ther..

[36] Johnstone L., Dallos R. (2013). Formulation in Psychology and Psychotherapy: Making Sense of People’s Problems.

[37] Schacht R.L., Dimidjian S., George W.H., Berns S.B. (2009). Domestic violence assessment procedures among couple therapists. J. Marital Fam. Ther..

[38] Nicholls T.L., Pritchard M.M., Reeves K.A., Hilterman E. (2013). Risk assessment in intimate partner violence: A systematic review of contemporary approaches. Partn. Abus..

[39] Statistics Norway. Family Counselling Service. https://www.ssb.no/en/sosiale-forhold-og-kriminalitet/barne-og-familievern/statistikk/familievern.

[40] Barne- likestillings- og inkluderingsdepartementet (2013). Tildelingsbrev til Barne-, Ungdoms- og Familiedirektoratet 2013.

[41] Barne- og likestillingsdepartementet (2016). Prop. 12 S (2016–2017). Opptrappingsplan mot Vold og Overgrep (2017–2021).

[42] Barne- ungdoms- og familiedirektoratet (2014). Dimensjonering og Organisering av Familieverntjenestene—En Evaluering.

[43] Justis- og beredskapsdepartementet (2013). Meld. St. 15 (2012–2013) Forebygging og Bekjempelse av Vold i Nære Relasjoner.

[44] Justis- og beredskapsdepartementet (2014). Et Liv uten Vold. Handlingsplan mot Vold i Nære Relasjoner 2014–2017.

[45] Royal Norwegian Ministry of Justice and Public Security (2012). Action Plan against Domestic Violence 2012.

[46] McLeod J. (2011). Qualitative Research in Counselling and Psychotherapy.

[47] Smith J.A., Flowers P., Larkin M. (2009). Interpretative Phenomenological Analysis: Theory, Method and Research.

[48] Smith J.A., Flowers P., Larkin M. (2022). Interpretative Phenomenological Analysis: Theory, Method and Research.

[49] Anderson N.E., Slark J., Gott M. (2019). Unlocking intuition and expertise: Using interpretative phenomenological analysis to explore clinical decision making. J. Res. Nurs..

[50] Blake S., Ruel B., Seamark C., Seamark D. (2007). Experiences of patients requiring strong opioid drugs for chronic non-cancer pain: A patient-initiated study. Br. J. Gen. Pr..

[51] Dunne E.A., Quayle E. (2001). The Impact of Iatrogenically Acquired Hepatitis C Infection on the Well-being and Relationships of a Group of Irish Women. J. Health Psychol..

[52] Flowers P., Knussen C., Duncan B. (2001). Re-appraising HIV testing among Scottish gay men: The impact of new HIV treatments. J. Health Psychol..

[53] Tomkins L., Eatough V. (2010). Reflecting on the use of IPA with focus groups: Pitfalls and potentials. Qual. Res. Psychol..

[54] Palmer M., Larkin M., De Visser R., Fadden G. (2010). Developing an interpretative phenomenological approach to focus group data. Qual. Res. Psychol..

[55] Smith J.A., Nizza I.E. (2021). Essentials of Interpretative Phenomenological Analysis.

[56] Reid K., Flowers P., Larkin M. (2005). Exploring lived experience. Psychologist.

[57] Love B., Vetere A., Davis P. (2020). Should Interpretative Phenomenological Analysis (IPA) be Used With Focus Groups? Navigating the Bumpy Road of “Iterative Loops,” Idiographic Journeys, and “Phenomenological Bridges”. Int. J. Qual. Methods.

[58] Dallos R., Vetere A. (2005). Researching Psychotherapy and Counselling.

[59] Schon D.A. (1991). The Reflective Practitioner: How Professionals Think in Action.

[60] Bateson G. (2000). Steps to an Ecology of Mind: Collected Essays in Anthropology, Psychiatry, Evolution, and Epistemology.

[61] Schön D.A. (1993). Generative metaphor: A perspective on problem-setting in social policy. Metaphor Thought.

[62] Gadamer H.G. (2004). EPZ Truth and Method.

[63] Ahmed S. (2014). The Cultural Politics of Emotion.

[64] Butler J. The Compass of Mourning. https://www.lrb.co.uk/the-paper/v45/n20/judith-butler/the-compass-of-mourning.

[65] Vetere A., Sheehan J. (2017). Supervision of Family Therapy and Systemic Practice.

[66] Thompson S., Thompson N. (2018). The Critically Reflective Practitioner.

[67] Tuval-Mashiach R. (2017). Raising the curtain: The importance of transparency in qualitative research. Qual. Psychol..

